# The Complementary and Alternative Medicine for Endometriosis: A Review of Utilization and Mechanism

**DOI:** 10.1155/2014/146383

**Published:** 2014-02-19

**Authors:** Sai Kong, Yue-Hui Zhang, Chen-Fang Liu, Ilene Tsui, Ying Guo, Bei-Bei Ai, Feng-Juan Han

**Affiliations:** ^1^Department of Obstetrics and Gynecology, Heilongjiang University of Chinese Medicine, Harbin 150040, China; ^2^Department of Obstetrics and Gynecology, The First Affiliated Hospital of Heilongjiang University of Chinese Medicine, Harbin 150040, China; ^3^Center for Post-Doctoral Studies, Heilongjiang University of Chinese Medicine, Harbin 150040, China; ^4^Pennsylvania State University College of Medicine, Hershey, PA 17033, USA

## Abstract

Endometriosis (EM) is one of the common gynecological conditions causing menstrual and pelvic pain and affects 10%–15% of women of reproductive age. In recent years, the complementary and alternative medical (CAM) treatment for EM has become popular due to the few adverse reactions reported. The CAM therapy for EM includes several different treatments such as herbs (herbal prescription, extract, and patent), acupuncture, microwave physiotherapy, and Chinese herb medicine enema (CHM enema). These CAM therapies are effective at relieving dysmenorrhoea, shrinking adnexal masses, and promoting pregnancy, with less unpleasant side effects when compared to hormonal and surgical treatments. In this review, we focus on the status quo of CAM on EM and try to identify therapeutic efficacy and mechanisms based on some clinical and experimental studies. We hope to provide some instructive suggestions for clinical treatment and experimental research in the future.

## 1. Introduction

**Figure 1:**
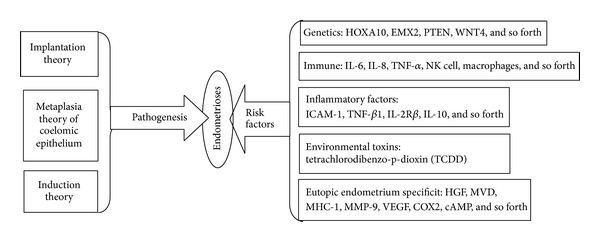
Pathogenesis and risk factors of endometriosis.

Endometriosis (EM) is a chronic, estrogen-dependent disorder and therefore generally occurs when endometrial tissue grows abnormally and adheres outside of the uterus. EM has a high prevalence rate in women of reproductive age and is divided into ovarian EM, peritoneal EM, and deep infiltrating EM according to the sites of implantation. The most common site is the ovary and the most common symptom is chronic pelvic pain, notably dysmenorrhoea, dyspareunia, and infertility, which all may lead to a reduction in the patient's quality of life. EM rarely undergoes malignant transformation, but with it there is a rising risk of ovarian, breast, and other cancers as well as autoimmune and atopic disorders [1]. The exact pathogenic mechanisms of EM remain unknown since Von Rokitonsky proposed the term “EM” in 1860 [2]; though recently, researchers propose several theories, including implantation theory, coelomic epithelium metaplasia theory, and induction theory. Research continues to examine other risk factors which may be potentially involved in the formation of EM, including genetics [3], immune factors [4], inflammatory factors [5], eutopic endometrium specificity, and environmental toxins [6] (Figure 1).

Treatment for EM can be medical and/or surgical. In western medicine, hormone replacement therapy is commonly used and involves oral contraceptives, progestogenics, gestrinone, Danazol (androgen derivates), and gonadotropin-releasing hormone (GnRH) agonists. Current investigations are also evaluating the role of GnRH antagonists, estrogen receptor beta (ER*β*) agoinist, progesterone receptor modulators, angiogenesis inhibitors, aromatase inhibitors, COX-2 (Cyclooxygenase 2) selective inhibitors, and immune modulators [7]. However, long-term administration with these therapies remains challening due to the plethora of serious adverse effects involved, such as massive hemorrhage, perimenopausal stage symptoms, masculinizing manifestation, and liver dysfunction. Data from the Cleveland Clinic showed that EM recurrence rate ranged between 20 and 40 percent within five years following conservative surgery, unless patients reached menopause, or hysterectomy was performed [8]. With this in mind, it is important to continue looking for other strategies to treat EM that may result in less adverse side effects.

Complementary and alternative medicine (CAM), commonly designated as “other than” conventional medicine, differs from medical mainstream, is widely accepted as a kind of medical treatment, and encompasses all health systems, practices, and modalities and their accompanying beliefs, theories, and attitude of a particular culture or society in a given historical period, as defined in the 1995 CAM Research Methodology Conference. Most therapies of CAM can be considered as part of five broad classes: biological based approaches, energy therapies, alternative medical systems, muscle and joint manipulation, and mind body therapies [9]. In most Asian countries, CAM is historic and has been widely utilized since the 19th century, while utilization in western countries has continued to increase [10, 11].

CAM is usually suggested as available complementary therapies. Among CAM, the nonpharmacologic interventions can reduce pain and concomitant mood disturbance to increase quality of life by employing mind-body interventions [12]. In the western world, CAM is predominantly used to treat or prevent musculoskeletal conditions or other conditions associated with chronic or recurring pain [13]. Additionally, CAM therapies are often utilized, attempting to manage chronic pain [14]. All these features show that CAM is fit for pain alleviation. As EM is typically accompanied by chronic pelvic pain and dysmenorrhoea, CAM therapies could be an effective treatment strategy. The CAM therapies for EM and its mechanisms in the published literature mainly include herbal products, acupuncture, microwave physiotherapy, CHM enema, and psychological interventions (Table 1). This review will focus on the status quo of CAM therapies and mechanism on EM.

## 2. Herbal Products Treatment on Endometriosis

Herbal products typically involve the use of herbal formulae of between 10 and 20 separate herbal ingredients selected from a Materia Medica of several hundred common herbs that are prepared either as a boiled decoction, as dried herbal extracts, or taken as pills or capsules (patent). Tables 2, 3, and 4 list the most commonly used herbs for EM [15–22]. Researchers evaluated the potential application and mechanism of herbal products on EM from these aspects: (1) clinical parameters including endometriotic lesion size measured by ultrasound examination; (2) pelvic pain and dysmenorrhoea measured by determination of clinical pain visual analogue scale (VAS) method; (3) laboratory parameters including some related molecules with immune regulation, antiangiogenesis, anti-inflammatory, and cell proliferation suppression factors; (4) implanted endometrium size measured by weight and volume in EM models.

### 2.1. Herbal Decoction Therapies and Mechanisms on Endometriosis

There are several common decoctions used to treat EM in China, including Xuefu Zhuyu decoction (XZD), Xiaochaihu decoction (XCHD), Qu Yi Kang (QYK), Yi Wei Ning (YWN), Yi Wei San (YWS), and Huoxue Xiaoyi decoction (HXD). XZD originated from 19th century in China and has been widely used to treat EM since 1983 [23]. Some studies have shown that XZD can relieve dysmenorrhoea, shrink ectopic lesions, and promote fertility; the average therapeutic effectiveness has been greater than 90% throughout the past 30 years [24, 25]. A clinical trial showed that XZD could relieve dysmenorrhoea as effectively as Mifepristone tablets, with the total effective rate being 90% and 73% in the XZD group and Mifepristone group, respectively, after 3 months of treatment with a study group of 120 patients [26]. Similar results were observed with a clinical trial of 42 patients treated with XZD for 6 months. Results showed that 5 of the 10 EM patients with infertility were pregnant and that 25 of the 42 EM patients were relieved of their pelvic pain [27]. Another trial [28] showed that the endometriotic lesions' size was both significantly reduced in XZD group and Danazol group, the total effective rate was 95.2% and 75.0%, respectively, and 23.6% of patients were pregnant in the XDZ group.

*XCHD*. has been widely used to treat a variety of disorders in ancient China since the Han dynasty. Some studies showed that XCHD could reduce the serum estradiol (E2) level and decrease the expression of P450aromatase protein and COX-2 protein in endometriotic tissue in the rat EM model. XCHD may play a key role in selectively inhibiting the expression of aromatase protein and COX-2 protein in EM tissues [29]. It is a feature that increased expression of proinflammatory cytokines such as tumor necrosis factor alpha (TNF-*α*) and interleukin 8 (IL-8) in peritoneal fluid of both EM patient and animal model [30, 31]. TNF-*α* plays an important role in the severity of EM-related dysmenorrhoea [32]. In a rat model receiving XCHD treatment, the volume of endometriotic lesion was significantly reduced concurrently with a reduction in the levels of IL-8, TNF-*α*, and vascular endothelial growth factor (VEGF) within serum and peritoneal fluid [33, 34]. Some researchers showed that XCHD could directly inhibit the growth of the ectopic endometrium in rat models by increasing Fas protein expression and promoting apoptosis in ectopic endometrial tissues [35–37].

*QYK*. is an empirical recipe which could alleviate the symptom of dysmenorrhoea by up to 90% and shrink the endometriotic lesion in up to 86.67% of patients with EM [38]. IL-2 and IL-6 play an important role in the cytokine network; the level of IL-2 reflects changes of the immune function; the level of IL-6 leads to local pelvic adhesions, fibrosis, and immunological abnormalities by mediating immune and inflammatory responses, thus contributing to the development of EM. Some studies found that QYK could reduce the levels of IL-2 and IL-6 in peritoneal fluid, inhibit the growth and proliferation of endometriotic tissue in rat EM models, and eliminate the new vascular networks on the surface of ectopic endometrium by reducing the expression of VEGF protein within the endometriotic tissue of the rat EM models. Furthermore, QYK could obviously reduce the estrogen receptor (ER) protein expression within endometrium and ectopic endometrium tissues, decreasing the amount of endometrium glands and reducing the endometriotic lesion of rat EM models [39–44].

One study showed that YWN can significantly shrink the endometrial lesion and relieve dysmenorrhoea by up to 93.48% for EM patients [45]. Some researchers indicated that YWN could facilitate apoptosis and inhibit proliferation of ectopic endometrium by improving the expression of Fas/FasL (Fas ligand) gene and B-celllymphoma-2 (Bcl-2) associated X protein (bax), reducing the expression of Bcl-2 and Cyclo-oxygenase-2 (COX-2) mRNA in endometriotic tissues of animal EM models [46–48].

Some studies indicated that serum 6-keto-prostaglandin Fl*α* (6-keto-PGF1*α*) and Thromboxane B2 (TXB2) within plasma were higher in endometriotic tissues than normal tissues, with 6-keto PGF1*α* inducing the severe dysmenorrhoea. YWS can decrease the levels of TXB2 and 6-keto-PGF1*α* within plasma in the endometriotic tissues, improving blood rheology and reducing the level of vasoactive substances as Danazol [49–52]. ICAM-1, an adhesion molecule of the immunoglobulin superfamily, plays a key role in regulating cell adhesion. Abnormal expression of ICAM-1 can promote the growth and metastasis of endometrial cells. In addition, MMP-9 also takes part in the regulation of cell adhesion and neovascularization [53]. HXD may downregulate the pathway of adhesion-aggression-angiogenesis, inhibiting the adhesion, aggression, and local angiogenesis by decreasing the expressions of ICAM-1, MMP-9, and VEGF. HXD exhibited a preferable and durable efficacy on recurrent EM treatment in rat models [54, 55].

*Other Herbal Decoction Therapies and Mechanisms on Endometriosis*. In China, several herbal mixtures are used to treat EM every year and demonstrate positive reports. For example, Cai Shi Nei Yi Fang could decrease the levels of IL-2 in the peritoneal fluid of rat EM models, while decreasing the levels of TNF-*α* in serum of rat EM models, thereby playing a role in immune regulation [56]. After treatment with Neiyi Zhitong decoction, the endometriotic lesions of the rat EM models were significantly reduced, with individual lesions minimized to only traces; the weight and volume of ectopic endometrium in rats were also reduced [57]. Neiyi Zhitong decoction could also reduce the levels of serum CA125 and regulate serum prostaglandin F2a (PGF2a), prosta-glandin2 (PGE2) concentration, alleviating dysmenorrhoea [58]. Fuzheng Huayu decoction has also been shown to be comparable to western tables in reducing EM symptoms as the total effective rate was up to 73.33% in one study compared with 76.67% for Gestrinone capsules [59]. Huazhuo Jiedu Huoxue Recipe may decrease plasma Orphanin FQ (OFQ), PGF2*α*, and PGE2 contents, thereby alleviating dysmenorrhoea and pelvic pain [60]. After 6 months of treatment with Yi Kun decoction, the total effective rate of the 92 EM patients was 91%, and 92% in the Danazol control group [61]. Juan Tong Yin lowered the level of IL-6 in ascitic fluid of white rabbit EM models, regulating the immune function, so as to improve the pelvic microenvironment, thus helping to prevent EM symptoms [62]. Xianji Pill did well in reducing the severity of dysmenorrhoea and shortened the frequency of dysmenorrhoea through the observation of 70 EM patients in one study; the total effective rate was 87.14% and 67.19% in the Xianji Pill treatment group and Danazol treatment group [63]. Wenshen Xiaozheng Tang (WXT) suppressed the growth of endometriotic lesions, partially through its antiangiogenic activity by lowering vascular density and reducing mRNA expression of hypoxia inducible factor-1*α* (HIF-1*α*) of the endometriotic lesions, decreasing concentration of VEGF in peritoneal fluid of WXT-treated rats [64]. WXT could also inhibit the production of proinflammatory cytokines and regulate the expression of invasion-related genes in the endometriotic lesions, thereby suppressing the development of EM [65]. As there are various herbal mixtures, only the most commonly used ones are discussed in this section.

### 2.2. Herbal Extracts Therapies and Mechanisms on Endometriosis

In recent years, several herbal extracts have been commonly used to EM treatment in China, including tripterygium wilfordii polyglycoside (Twp), puerarin, turmeric, reseratrol, green tea epigallocatechin-3-gallate (EGCG), ginsenoside Rg3, and so on. Tripterygium Wilfordii polyglycoside (TWP) is an herbal extract derived from Tripterygium, a Chinese herb, and made into oral TWP tablets. TWP is widely used to treat autoimmune diseases, such as rheumatoid arthritis (RA), ankylosing spondylitis (AS), and nephrotic syndrome (NS) in China [66–70]. TWP is also frequently used for EM treatment in China [71, 72]. A study of retrospective analysis in 40 EM patients with TWP treatment from 1986 to 1990 showed that 18 cases were very effective, 17 cases were mildly effective, and 5 cases were ineffective with the therapeutic overall effective rate up to 87.5% [73]. Another study found that dysmenorrhoea and menstrual disorder of 97.5% of EM patients with Tripterygium treatment after 3 months were significantly improved and the serum CA125 level was decreased [74]. One research study indicated that the average volume of endometrial implants was significantly reduced, in addition to the endometrial antibody (EMAb) level which was decreased and the changed gonadotropic cells (G-cell) morphology which gradually disappeared in a rabbit EM models treatment with TWP [75].

*Curcumin*. is a polyphenolic monomer extract from Turmeric, a Chinese herbal medicine which activates microcirculation and possesses various pharmacological activities including anti-inflammatory, antioxidant, and antiproliferative components. In addition, Curcumin may exert beneficial effects on the motor coordination of adolescent rats exposed to ethanol [76]. Both Curcumin and the Chinese medicine formula with Turmeric may significantly alleviate and improve the symptoms of EM patients [77, 78]. Some studies discovered that the number of microvessels and the protein expression of VEGF were decreased in the ectopic endometrium of rat EM models with Curcumin treatment [79], and the mRNA expression of the tumor necrosis factor-*α*-induced (TNF-*α*-induced) cell surface and total protein expression of intercellular adhesion molecule-1 (ICAM-1) and vascular cell adhesion molecule-1 (VCAM-1) were decreased. Additionally, the secretion of IL-6, IL-8, and monocyte chemotactic protein-1 (MCP-1) was decreased and the activation of transcription factor NF-*κ*B was suppressed in human endometriotic stromal cells with Curcumin treatment [80]. Some other studies showed that the expression and activities of matrix metalloproteinase (MMP)-2 [81], MMP-3 [82], MMP-9 [83], and VEGF [84] protein were inhibited in rat EM models that received Curcumin treatment, and the endometriotic lesions were reduced.

*Puerarin*. is a major isoflavonoid compound extracted from Radix puerariae, a Chinese herbal medicine, which has a potential weak estrogenic effect by binding to estrogen receptors (ERs) [85, 86]. Some studies showed that the weight of endometriotic tissue and the level of serum estrogen were lower in Puerarin and Danazol treatment group than control group at rat EM models [87]; the levels of MMP-9, ICAM-1, and VEGF protein were reduced, while the tissue inhibitor of metalloproteinase-1 (TIMP-1) level was increased in endometriotic stromal cells (ESCs) with Puerarin treatment [86]. The invasion of endometriotic tissue is dependent on MMPs and TIMPs, as they both play a key role in growth and decomposition of endometrium tissue. The VEGF family is the most important in angiogenesis which contributes to the growth and invasion of ectopic endometrium. Puerarin may suppress invasion of ESCs and the vascularization of ectopic endometrial tissues by regulation of MMP-9, ICAM-1, VEGF, and TIMP-1.

*Resveratrol.* is a polyphenol which is mainly extracted from grapes, *Polygonum cuspidatum*, peanuts, mulberry, and other plants. Resveratrol could improve the efficiency of reprogramming mouse embryonic fibroblasts into induced pluripotent stem cells by mimicking hypoxia in cells at a low concentration (10 *μ*mol/L) [88]. Resveratrol has been proposed to treat EM as natural medicine due to its strong antioxidant properties. A research reported that Resveratrol potentiates the effect of oral contraceptives in the alleviation of EM-associated dysmenorrhoea with EM patients by inhibiting aromatase and COX-2 expression in the endometrium [89]. Some studies indicated that the number of endometrial implants decreased by 60% and the total volume of lesions was reduced by 80% in rat EM models with Resveratrol treatment [90]; the expression of VEGF was lower in the endometriotic tissue and peritoneal fluid, at the same time, the level of monocyte chemotactic pretein 1 (MCP-1) was also lower in the endometriotic tissue in rat EM models with Resveratrol treatment [91]. Furthermore, Resveratrol inhibited angiogenesis in peritoneal and mesenteric endometriotic lesions by significantly reducing microvessel density and proliferating activity of CD31-positive endothelial cells in the newly developing microvasculature of the lesions in rat EM models treated with Resveratrol for 4 weeks, while at the same time, the growth rate was lower and the final size was smaller within lesions in Resveratrol-treated mice than controls due to lower numbers of proliferating cell nuclear antigen- and Ki67-positive stromal and glandular cells [92].

*Green Tea Epigallocatechin-3-Gallate (EGCG)*. is a catechin monomer extracted from Green tea, the major constituent of which is tea polyphenols. EGCG plays a key role in antioxidation and antiangiogenesis, enhancing apoptosis and inhibiting function of microvessels in the lesions, thereby reducing the size and the weight of lesions and inhibiting the development and growth of experimental EM [93]. What is more, EGCG selectively suppresses the expression of vascular endothelial growth factor C (VEGFC) and tyrosine kinase receptor VEGF receptor 2 (VEGFR2) and reduced VEGFR2 and ERK activation in endothelial cells [94]. Another experiment reported that after treatment with EGCG for 2 weeks, endometriotic lesions and glandular epithelium were smaller or eccentrically distributed by downregulating angiogenic vascular endothelial growth factor A (VEGFA) mRNA levels and upregulating nuclear factor kappa B (NF*κ*B) and mitogen activated protein kinase 1 (MAPK-1) mRNA levels in lesions [95]. Furthermore, Laschke et al. found that EGCG could reduce E2-stimulated activation, proliferation, and VEGF expression of endometrial cells in rat EM models, thus preventing the formation of new endometriotic lesions [96].

*Ginsenoside Rg3*. is found exclusively in the plant genus *Panax*, a kind of steroid glycosides, and triterpene saponins, which inhibits antioxidative, anti-inflammatory, and matrix metalloproteinase activities. A clinical observation showed that ginsenoside Rg3 achieved equivalently high efficacy and fewer side effects as compared with gestrinone. Ginsenoside Rg3 could be utilized as clinical medication for EM patients [97]. Furthermore, an experiment observed that the volume of endometriotic lesions was reduced and the microvessel density (MVD) expression was lower in ectopic tissues within ginsenoside Rg3 treatment group than in the gestrinone group. In addition, ginsenoside Rg3 exhibited an antiangiogenic effect by inhibiting the expression of inhibitors of DNA binding 1 (ID-1) gene and neuropilin-1(NRP1) gene in rat EM models [98–101]. 

*Other Herbal Extracts for Endometriosis Treatment*. Herbal extracts are a hot topic in Chinese medicine development with new extracts constantly being investigated; though many reports are not thorough enough to show the mechanism of their treatment for EM. Artemisinin could increase the apoptosis index of the ectopic endometrium by decreased Bcl-2 protein and MVD, thereby significantly decreasing the size of implants in endometriotic rat models as compared to Danazol treatment groups [102]. Querce decreased the size of implants in the rat EM models by reducing the expression of heat shock protein 70 (HSP 70) and VEGF as similar to Danazol treatment group [103].

### 2.3. Herbal Patent Medicine Therapies and Mechanisms on Endometriosis

In the past 10 years, more than 30 herbal patents have been commonly used to treat EM patients in clinic including capsule, pill, and liquid. These patents are made up of several herbs by thousands clinical experiences. Herbal patent is more convenient than the decoction due to modified formulation and is therefore also more suitable for quantitative study. However, the studies about herbal patents in the areas of in-depth mechanism are far less than the herbal decoctions. Some findings about the mechanisms of several commonly used patents were listed as follows.

Some researches indicated that Guizhi Fuling Capsules (GZFLC) was effective for EM treatment. A 3-month clinical observation with 48 EM patients showed that the effective rate was 83.3% and 87.5% in GZFLC group and Mifepristone group, respectively. Additionally, the level of serum CA125 was decreased from (94.45 ± 23.26) U/mL and (96.45 ± 24.13) U/mL to (46.47 ± 11.28) U/mL and (43.67 ± 12.11) U/mL in GZFLC group and Mifepristone groups, respectiely, while the negative conversion rate of endometrial antibody (EMAb) was 65.0% and 69.2%, respectively [104]. A study showed that the volume of lesions in rat EM models was reduced from (331.40 ± 158.12) mm^3^ to (50.32 ± 33.28) mm^3^ after treatment with GZFLC for 4 weeks and the percentage of some immunocytes was significantly increased including CD+3, CD+4, CD+4/CD+8, and NK cells in rat EM models, indicating that GZFLC plays an important role in suppressing endometriotic implants by regulating the immune function [105, 106].

Sanjie Zhentong Capsules (SZC) is commonly used to treat gynecological tumors, including EM. A random single-blind clinical observation showed that the symptoms of 92.9% and 77.5% patients were alleviated, respectively, in the SZC treatment and the Danazol treatment of 112 EM patients [107]. After laparoscopic surgery, the post-2-year recurrence rates were, respectively, 14.0% and 13.3% in SZC treatment group and Danazol treatment group, while being 35.0% in the control group, indicating that SZC could reduce the post-2-year recurrence rate after surgical treatment [108]. However, we have not found any scientific data about the mechanism of SZC in EM animal models.

Other clinical observations indicated that the total effective rate was, respectively, 84% and 86% in the Yiweikang Granules (YWKG) treatment group and the Danazol treatment group with 50 EM patients, with the levels of CA125 and VEGF protein in both groups significantly reduced to the same levels after treatment. Furthermore, YWKG could significantly inhibit the growth of ectopic endometrium [109]. Another observation of 100 EM patients showed that YWKG treatment had functions of diminishing ectopic lesion, releasing pelvic pain, improving haemorheology and pelvic blood flow, and the level of serum estradiol (E2) was lowered from (1458.4 ± 183.5) pmol/L to (1202.0 ± 153.1) pmol/L after YWKG treatment [110]. The negative conversion rate of EMAb was 66.7% with Dahuang Zhechong Wan (DHZCW) and 75% with Danazol within EM patients. Both DHZCW and Danazol could relieve the clinical symptoms; the total effective rate was 80.8% and 86.7%, respectively [111]. DHZCW could reduce the levels of serum E2 and prolactin (PRL) and increase the serum progesterone (P) level in rat EM models [112]. Yang found that Xiang Leng Pill (XLP) could reduce the level of serum CA125 from (93.4 ± 3.6) IU/L to (48.4 ± 2.4) IU/L after 3-month treatment and relieve symptoms of dysmenorrhoea measured with determination of clinical pain visual analogue scale (VAS) [113]. Another study showed that XLP decreased the levels of interleukin-8 (IL 8) and tumor necrosis factor *α* (TNF-*α*) in peritoneal fluid and peripheral blood as did Danazol treatment in rat EM models [114].

## 3. Acupuncture and Moxibustion Treatment on Endometriosis

Acupuncture and Moxibustion treatment is one of the traditional Chinese practices widely used in China and some Asian countries. In China, practitioners of acupuncture and moxibustion regard the human body as a whole based on the theories of meridian, viscera, and Qi-Blood (Chinese medicine believes that Qi is the most basic form of human material foundation, human life, and activities. Qi and Blood have their different roles but are interdependent with each other, supplying nutrients for the organs and tissues to maintain life activities). Meridian in traditional Chinese medicine (TCM) refers to the pathways of Qi and Blood, and those pathways are interconnected with each other. As a treatment therapy, acupuncture has been used as medical methods since antiquity in China. Currently, it has become an acceptable therapy under strict regulation and has gained a license in 40 states [115]. Gradually, acupuncture has become accepted as a CAM therapy by the United States National Institutes of Health, the National Health Service of the United Kingdom, and the World Health Organization [116, 117]. As an alternative or complement therapy, Acupuncture and Moxibustion treatment are effective for EM; though a lack of systematic review for these therapies still remains in the scientific literature. This paper will systematically review the utilization and mechanism of Acupuncture and Moxibustion treatment for EM. We found that the acupoint of Ren Meridian (RN) and Spleen Meridian of Foot-Taiyin (SP) is more predominately involved in the Acupuncture and Moxibustion treatment than that of Bladder Meridian (BL) and Kidney Meridian (KI). The main acupoints and minor acupoints for the treatment of EM in Chinese reports are listed in Table 5.

### 3.1. Acupuncture Treatment on Endometriosis

Acupuncture therapy includes needling, auricular point, and moxa-moxibustion. It has the function of dredging meridian, regulating the balance of Yin and Yang (Chinese medicine believes that the balance of Yin and Yang determines people's health), enhancing “Qi-Blood” circulation, thereby strengthening body's resistance to disease and eliminating pathogenic factors. In clinic, acupuncture therapy for EM is confirmed to improve efficacy with fewer side effects, especially in EM-associated dysmenorrhoea. Acupuncture analgesia is usually used to treat pelvic pain and dysmenorrhoea related to EM by mediating the central nervous system (CNS) and releasing some specific neurotransmitters. Zeng and Hong [118] used warm acupuncture (needle warming through moxibustion) and ordinary acupuncture to treat 40 EM patients, respectively, with the same acupoint. Both of these two methods were effective and the effective rate was 95.0% and 77.5%, respectively. The acupoints used were Guanyuan (RN4), Zhongji (RN3), Tianshu (ST25), Sanyinjiao (SP6), Taichong (LR3), and Zusanli (ST36). Chen and Lin [119] treated a total of 70 patients of EM-related dysmenorrhoea with abdominal acupuncture or Danazol. The acupoints used were Zhongwan (RN12), Xiawan (RN10), Qihai (RN6), Guanyuan (RN4), Zhongji (RN3), and Wailing (ST26), and the treatment was continued for three consecutive menstrual cycles. Results showed that the total effective rate was 91.4% and 80.0%, respectively. Liu et al. [120] treated 35 EM patients with existing dysmenorrhea with the acupoints of Qihai (RN6), Guanyuan (RN4), Qixue (KI13), Dahe (KI12), Zigong (EX-CA1), Diji (SP8), and Taixi (KI3). Results showed that the total effective rate was 77.14% after treatment for three menstrual cycles. One experiment study showed that both acupuncture and Danazol significantly reduced the level of TNF-*α* in peritoneal fluid of EM rat model [121].

### 3.2. Moxibustion Treatment on Endometriosis

Moxibustion originated from the Spring and Autumn Periods and the Warring States Periods in ancient china and has been popular since that time. Moxibustion is a technique which applies heat to acupoints by burning compressed powdered herbal material at the acupoints to stimulate them.

Moxibustion includes moxibustion with moxa cone, moxa stick, and herbal medicine cake, burning rush moxibustion and crude herb moxibustion. Moxibustion with moxa cone or moxa stick is most commonly utilized. Chinese medicine believes that the Moxibustion could warm meridians, relieve pain, and promote blood circulation. Chiu [122] analyzes the factors about function of Moxibustion and demonstrates that with the exception of the temperature-related factors, there are some nontemperature-related factors, including smoke effects, herbal effects, and biophysical effects (far infrared). She and Xiong [123] treated 20 EM patients of dysmenorrhoea using Guanyuan (RN4), Zhongji (RN3), Qihai (RN6), Tianshu (ST25), Sanyinjiao (SP6), Zigong (EX-CA1), and Ashi points with Ginger Moxibustion. The total effective rate was 95.0%, and following six menstrual cycles, the symptoms of dysmenorrhoea did not recur within 18 patients. Several animal experiments showed that Moxibustion with herbal medicinal cake could treat EM by reducing the levels of serum IL-6 and plasma PGE2 and increasing the level of plasma 6-keto-PGF1*α* [124–126].

### 3.3. Acupuncture Combined with Moxibustion Treatment on Endometriosis

In the clinic, practitioners usually utilize acupuncture and moxibustion together to treat EM. Chen et al. [127] reported that 72 EM patients were treated with acupuncture and moxibustion therapy, 42 patients were pregnant, and the total effective rate was 93.05%. In another observation of 42 cases, EM patients were given techniques of acupuncture combined with moxibustion and results showed that the total curative effective rate was 92.86% [128]. Combined with acupuncture, moxibustion treatment can effectively arouse the regulating function of meridian, thereby improving the body's immune function to relieve symptoms of EM.

### 3.4. Acupoint Injection Treatment on Endometriosis

Acupoint injection is also known as “water injection” since Chinese herbal and western medicine are injected into acupoints to treat disorders. It is based on the same meridian theory for acupuncture. This therapy collects acupuncture, medicine and meridian effects together. Lin et al. [129] treated rat EM models with acupoint injection. Results showed that acupoint injection with low doses of Alarelin can have an analgesic effect on rat EM models and produce a better effect than intramuscular injection with Alarelin. Sun [130] found that the pelvic mass of ovarian chocolate cysts was significantly reduced in 88.6% EM patients after treatment by acupoint injection with Chinese herbal mixture, and the acupoints were Sanyinjiao (SP6), Xuehai (SP10), and Zigong (EX-CA1).

### 3.5. Acupuncture Combined with Chinese Medicine to Treat Endometriosis

In clinic, combining acupuncture and Chinese medicine for EM treatment could achieve better effect through internal recuperate and external stimulation. A study observed 48 patients of EM-associated dysmenorrhoea treated with acupuncture and “Quyu Jiedu Xiaozheng decoction (a kind of Chinese herbal medicine).” The results indicated that the total curative effective rate was 92.0% [131]. In another clinical observation of 58 EM patients treated with “Shaofu Zhuyu decoction (a kind of Chinese herbal medicine)” combined with acupuncture for three months, results showed that the symptom of dysmenorrhoea among all of the patients disappeared [132]. Zhang [133] treated 53 EM patients by acupuncture and Chinese medicine, the total effective rate was 87.09%, and the pregnancy rate was 40%. The acupoints were Zigong (EX-CA1), Sanyinjiao (SP6), Ligou (LR5), Taichong (LR3), Guanyuan (RN4), and Guilai (ST29). Another clinical observation of EM showed that after treatment with acupuncture and Chinese herbal medicine, the total effective rate was 100% [134]. Fu and Xia [135] found that after treatment with acupuncture and medicine, the levels of serums CA125, PGE2, and PGF2*α* were lower, while the serum *β*-EP level was higher than before in EM patients. Chen et al. [136] took the “rat models” acupoint brief as standard to select acupoint [137], treated the EM rat model with acupuncture and Chinese herbal medicine, and found that the volume of the endometriotic implants were reduced and the expression of MMP-2 protein in the ectopic endometrium was increased.

### 3.6. Electroacupuncture Treatment on Endometriosis

Electroacupuncture (EA) therapy is a new acupuncture therapy that gives the body a bioelectrical trace current after needling into the acupoint. The advantage of EA is that it can control the stimulation for a long time, adjust the physiological functions of the human body, promote blood circulation, adjust muscle tension, and so forth. EA is commonly used to relieve symptoms of pain, arthralgia, and organ dysfunction in clinic. 80 cases of EM patients were randomly divided into ear EA group and body EA group. The symptoms of dysmenorrhoea were significantly relieved, and the plasma PGE2 level was decreased, while the plasma 6-Keto-PGF1*α* level was increased in both the ear EA and body EA groups [138]. An experiment [139] indicated that EA in the ear acupoints could have an analgesic effect by raising the beta-Endorphin (*β*-EP) protein level of the hypothalamus and pituitary in EM rabbit model. Another experiment also found that EA in Guanyuan (RN4) and Diji (SP8) had an analgesic effect on EM rat models by improving the level of *β*-EP and Dynorphin protein in the hypothalamus and pituitary of rat EM models [140].

### 3.7. Acupoint Sticking Treatment on Endometriosis

Acupoint sticking therapy is a noninvasive acupoint therapy via sticking the acupoints with some specially modulated medication, based on the Chinese meridian theory. Acupoint sticking therapy is easy for patients to use themselves, so it is accepted as an adjuvant therapy to the treatment of dysmenorrhoea. There are few research studies about acupoint sticking treatment on EM. The medication typically consists of herbal powder mixed with water, vinegar, wine, egg white, honey, vegetable oil, cool oil, liquid, or saliva. The mechanism of the acupoint sticking therapy is complex and not completely understood yet. Some researchers showed that sticking on acupoint can strengthen drug's absorption and recuperate meridian disorder by directly stimulating acupoint with herbal medicine. An experimental study on EM rat models showed that both sticking with Resina Draconis cataplasma in the Guanyuan (RN4) acupoint and sticking with cataplasma in the same acupoint could inhibit the growth of EM and promote glandular atrophy. It may show its effect by reducing the levels of plasma TXB2 and 6-keto-PGF in rat EM models [141].

### 3.8. Auricular Acupoint Treatment on Endometriosis

Chinese medicine practitioners believe that acupoint on the ear can reflect the general health of the human body. Certain parts of the human body that are unwell will be apparent within certain auricular areas. Auricular acupoint therapy is applied for the situations of pain, inflammatory diseases, functional disorders, and endocrine and metabolic disorders. In China, auricular acupoint therapy is usually applied as adjuvant therapy with oral decoction or acupuncture and moxibustion therapies. Auricular acupoint therapy can be used as daily care treatment for EM, which is a convenient method to improve the patient's quality of life. Duan et al. [142] found that auricular acupressure could relieve uterine smooth muscle spasm through meridian induction and neurotransmission, reducing the secretion of serum PGE 2.

## 4. The Treatment of Chinese Herbal Enema and Microwave Physiotherapy for Endometrioses

### 4.1. The Treatment of Chinese Herbal Enema for Endometriosis

Chinese herbal enema is also known as anorectal drug delivery method which consists of pouring Chinese herbal medicine into the rectum where it remains for four to five hours to make the Chinese herbal medicine fully absorb through the intestinal mucosa to treat some specific diseases. This method can reduce the stimulation of the drug to alimentary canal and avoid the damage of the drug by digestive enzymes. Furthermore, Chinese herbal enema can improve the bioavailability of the drug and reduce the damage to the liver and other organs. There were several observations about treatment of EM with Chinese herbal enema which found that Chinese herbal enema has significant therapeutic effect, especially on reducing endometriotic lesions. Zhou [143] treated 64 EM patients with Chinese herbal enema (drugs: Common Burreed Tuber 10 g, Red Peony Root 12 g, Aeruginous Turmeric Rhizome 9 g, Turmeric Root Tuber 10 g, Peach Seed 10 g, Degelatined Deer-horn 12 g, Malaytea Scurfpea Fruit 9 g, Cassia Twig l0 g, Ground Beetle 8 g, inner membrane of chicken gizzard 15 g) and found that the total clinical effective rate was 93.8%. Wu [144] treated 51 cases of EM-associated infertility patients who were randomized into two treatment groups with Chinese herbal enema and Danazol treatment for 9 months (drugs: Common Burreed Tuber 10 g, Aeruginous Turmeric Rhizome 10 g, Sargentodoxa cuneata 15 g, Chinese Honeylocust Spine 15 g, Honeycomb 10 g, Red Peony Root 15 g, and Peach Seed 10 g). Results showed that the pregnancy rate was 42.4% and 27.8%, respectively, after followup of 26 months. Zhang and Zhu [145] treated EM patients with retention enema of traditional Chinese herbal medicine (drugs: Danshen Root, Bulb of Thunberg Fritillary, Frankincense, Myrrha, Sargentodoxa cuneata, Patrinia, Cassia bark, Yanhusuo Tuber, Red Peony Root, Chinese Angelica, and Peach Seed) or oral Danazol and the results showed that the effect of both groups was similar, while the adverse reactions were far less in the retention enema group than those of the Danazol group.

### 4.2. The Treatment of Microwave Physiotherapy on Endometriosis

Microwave is nonionizing radiation and ultrahigh frequency electromagnetic waves with frequency between 300 and 300000 MHz and the wavelength between 1 mm and 1 m. Before treatment with microwave physiotherapy, the patient's medical history is needed to assess the patient's condition. The microwave physiotherapy should be used only in the nonacute phase of EM; it should be used with caution if the patient has metal implants (such as women with infertility link) in the body. Ask the patients to take supine position and expose the lower abdomen. Put the microwave physiotherapy instrument facing patient's lower abdomen with the distance of 35–45 cm, depending on patient's skin temperature. 30 min is a course of treatment. Microwave physiotherapy works by absorbing surrounding material and producing a heating effect, without changing the chemical nature of the surrounding substance, and has high security [146]. Kanaoka Y found that microwave physiotherapy could adjust menorrhagia and inhibit uterus enlargement caused by adenomyosis [147]. As an adjuvant treatment for EM, microwave physiotherapy shows a synergistic effect which increases the absorption rate of Chinese herbal medicine.

### 4.3. Chinese Herbal Enema Combined with Microwave Physiotherapy for the Treatment of Endometriosis

Chinese herbal enema and microwave physiotherapy are always combined for the treatment of endometrioses as adjuvant therapy which can produce a heating effect to promote increased absorption of Chinese herbal enema via rectum mucosa. Chinese herbs formula used as enemas can be modified according to the various symptoms patients present with. Microwave treatment is utilized after Chinese herbal retention enema to strengthen the permeation of medication and promote the absorption and metabolism of inflammation and exudates. Since the rectum and pelvic organs are close to each other, herbal enema can be directly absorbed through the rectum and affect the lesion site directly [148]. Some clinical studies showed that Chinese herbal enemas combined with microwave physiotherapy could effectively relieve the symptoms of lower abdominal pain, shrink cysts, and increase the conception rate; the total effective rate was 94. 2%, the pregnancy rate was 45%, and the symptoms of dysmenorrhoea and menstrual disorder almost decreased entirely [149–151].

### 4.4. Chinese Herbal Enema Combined with Oral Chinese Medicine on Treatment of Endometriosis

Chinese herbal enemas combined with oral Chinese herbal medicine are the most commonly utilized treatment for EM patients who are treated in hospital, with the total effective rate varying between 83.8% and 93.88% [152–156].

## 5. Others CAM Therapies

There are some other therapies effective for EM in addition to these therapies mentioned above, such as hypnosis [157] and thermal biofeedback [158]. While there is lack of detailed data about the mechanisms, we will continue to focus on future avenues of research in this field.

## 6. Conclusions

CAM therapies utilized in patients with EM in the literature include herbs, acupuncture, CHM enema, microwave physiotherapy, and psychological intervention. None of these therapies are entirely curative for EM and neither can they fully eradicate the endometriotic lesions. These therapies may effectively modulate the progress of EM, however, by shrinking the lesions, suppressing the symptoms, and decreasing the recurrence rate. Although, CAM therapies have been gradually accepted in some countries, some obstacles—such as the lack of more thorough safety and efficacy studies—still hinder more widespread application of CAM therapies throughout the world. We found some mainly additional obstacles including: (1) selective publication of only positive results with varying study qualities and standards, (2) lack of large-sample sizes and randomized controlled trials, (3) and the lack of confirmatory animal studies with therapies such as auricular acupoint, Chinese herbal enema, microwave physiotherapy, and psychological intervention. The molecular mechanisms of some CAM therapies need to be further investigated and confirmed in the future.

In summary, the active principle of the CAM therapies has a strong scientific foundation and researchers are increasing their interest in this area of medical treatment. Standardizations of the effective CAM therapies are still needed, however, including managing the pharmaceutical form of herbal agents and controlling the quality of acupuncture methods in order to increase the benefits of these alternative medical interventions to patients with EM throughout the world.

## Figures and Tables

**Table 1:** General view of all therapeutic approaches.

Therapeutic approaches	Clinical indication	Specifications	Efficacy	Precautions
Herbal products	EM with chronic pelvic pain, dysmenorrhea, and infertility	According to TCM practitioners' judgment of the disease, propose appropriate TCM prescriptions	Alleviate dysmenorrhoea Shrink endometriotic lesion Promote pregnancy Reduce recurrence rate	Patients who are allergic to some foods and pollen should take the herbal products with caution

Acupuncture and Moxibustion	EM with chronic pelvic pain, dysmenorrhea, and infertility	Take the appropriate acupoints and choose needling, auricular point, or moxa-moxibustion therapy, according to the disease status of patient. 30 min is a course of treatment for acupuncture (needling, auricular point); 40–50 min is a course of treatment for moxibustion	Alleviate dysmenorrhoea Promote pregnancy	Some patients may occur fainting condition

CHM enema	EM with chronic pelvic pain, dysmenorrhea, and infertility	Ask the patient to take the left lateral decubitus position. Put the boiled TCM herbal liquid into 20 mL syringe, with the temperature of 38~40°C. With a disposable catheter connection, slowly push TCM herbal liquid into the rectum. Tell the patient to relax and keep the TCM herbal liquid more than 2 hours	Alleviate dysmenorrhoea Shrink endometriotic lesion	Unfit for predominant irritable bowel syndrome patients

Microwave physiotherapy	nonacute phase of EM	Ask the patient to take supine position. Put the microwave physiotherapy instrument facing patient's lower abdomen, with the distance of 35–45 cm. 30 min is a course of treatment	Alleviate dysmenorrhoea Shrink endometriotic lesion	Attention to operating time, adjusting the distance of microwave physiotherapy equipment, so as not to scald patients

**Table 2:** Ingredients of herbal preparations.

Herbal mixture	Ingredients
Xuefu Zhuyu decoction (XZD)	Chinese Angelica (9 g), Rehmannia Root (9 g), Peach Seed (12 g), Saffron (9 g), Orange Fruit (6 g), Red Peony Root (6 g), China Thorowax Root (3 g), Ural Licorice (6 g), Platycodon Root (4.5 g), Szechwan Lovage Rhizome (4.5 g), Twotoothed Achyranthes Root (9 g)
Xiaochaihu decoction (XCHD)	China Thorowax Root (30 g), Baical Skullcap Root (18 g), Ginseng (18 g), Pinellia Tuber (18 g), Ural Licorice (18 g), Fresh Ginger (18 g), Chinese Date (12 g)
Qu Yi Kang (QYK)	Chinese Angelica (12 g), Red Peony Root (15 g), Szechwan Lovage Rhizome (10 g), Yanhusuo Tuber (15 g), Common Burreed Tuber (10 g), Aeruginous Turmeric Rhizome (10 g), Frankincense (9 g), Myrrha (9 g), Cassia Twig (9 g), Danshen Root (15 g), Cattail Pollen (10 g), Draconis Resin (6 g), Tangerine Seed (10 g)
Yi Wei Ning (YWN)	Red Peony Root (20 g), China Thorowax Root (10 g), Aeruginous Turmeric Rhizome (15 g), Yanhusuo Tuber (15 g), Centipede (2), Baical Skullcap Root (15 g), Honeysuckle Flower (30 g), Coix Seed (20 g), Largeleaf Gentian Root (15 g), Oyster Shell (10 g)
Yi Wei San (YWS)	Draconis Resin (30 g), Sanchi (30 g), Coix Seed (240 g), Appendiculate Cremastra Pseudobulb (240 g), Myrrha (80 g), Danshen Root (120 g), Thunberg Fritillary Bulb (150 g), Red Peony Root (150 g)
Huoxue Xiaoyi decoction (HXD)	Danshen Root, Red Peony Root, Aeruginous Turmeric Rhizome

Patent medicine	Ingredients

Guizhi Fuling Capsules (GFC)	Ramulus Cinnamomi, Poria, Cortex Moutan, Radix Paeoniae Rubra, Semen Persicae
Sanjie Zhentong Capsules (SZC)	Resina Draconis, Radix Notoginseng, Fritillaria thunbergii, Semen Coicis
Dahuang Zhechong Wan (DZW)	Rhubarb (300 g), Ground Beetle (30 g), Leech (60 g), Gadfly (45 g), Northeast Giant Black Chafer (45 g), Dried Lacquer (30 g), Peach Seed (120 g), Bitter Apricot Kernel (120 g), Baical Skullcap Root (60 g), Rehmannia Root (300 g), White Peony Root (120 g), Ural Licorice (90 g)
Yiweikang Granule (YWKG)	Milkvetch Root (15 g), Cassia Twig (10 g), Peach Seed (10 g), Saffron (10 g), Yanhusuo Tuber (10 g), Leech (5 g), Szechwan Lovage Rhizome (10 g), Immature Tangerine Peel (10 g), Trogopterus Dung (10 g), Cattail Pollen (6 g), Common Fennel (10 g), Combined Spicebush Root (10 g)
Xiang Leng Wan (XLW)	Peach Seed (12 g), Common Burreed Tuber (10 g), Aeruginous Turmeric Rhizome (10 g), Tree Peony Root Bark (12 g), Red Peony Root (12 g), Cassia Twig (12 g), Common Aucklandia Root (12 g), Immature Tangerine Peel (12 g), Orange Fruit (12 g), Szechwan Chinaberry Fruit (12 g), Common Fennel (10 g), Indian Bread (12 g)

TCM enema	Ingredients

Private customized TCM enema decoction	Common Burreed Tuber (10 g), Red Peony Root (12 g), Aeruginous Turmeric Rhizome (9 g), Turmeric Root Tuber (10 g), Peach Seed (10 g), Degelatined Deer-horn (12 g), Malaytea Scurfpea Fruit (9 g), Cassia Twig (10 g), Ground Beetle (8 g), inner membrane of chicken gizzard (15 g)
Private customized TCM enema decoction	Common Burreed Tuber (10 g), Aeruginous Turmeric Rhizome (10 g), Sargentodoxa cuneata (15 g), Chinese Honeylocust Spine (15 g), Honeycomb (10 g), Red Peony Root (15 g), Peach Seed (10 g)
Qing Yi decoction	Danshen Root (20 g), Bulb Thunberg Fritillary (10 g), Frankincense (6 g), Myrrha (6 g), Sargentodoxa cuneata (30 g), Patrinia (15 g), Cassia bark (6 g), Yanhusuo (12 g), Tuber (12 g), Red Peony Root (12 g), Chinese Angelica (12 g), Peach Seed (10 g)

Acupoint injection TCM	Ingredients

Xiao Qiao decoction	Danshen Root (20 g), Common Burreed Tuber (10 g), Aeruginous Turmeric Rhizome (10 g), Chinese Lobelia (20 g), Spreading Hedyotis (20 g), Baikal Skullcap (30 g), Bulb Thunberg Fritillary (10 g), Oyster Shell (30 g), Yanhusuo (10 g), Teasel (10 g), Shorthorn Barrenwort (10 g), Honeylocust Spine (30 g)

**Table 3:** Herbal mixture for EM treatment in the literature.

Herbal mixture; sample/case number (*n*)	Control; sample number (*n*)	Total clinical effect rate	Model used	Therapeutic effects and actions	References
Xuefu Zhuyu decoction (XZD); *n* = 79	Gestrinone Capsule; *n* = 56	*T*: 91.14% versus 91.07%	Human study	Alleviate dysmenorrhoea Shrink endometriotic lesion	[25]
XZD; *n* = 60	Mifepristone Tablets; *n* = 60	*D*: 90.0% versus 70.0%	Human study	Alleviate dysmenorrhoea	[26]
XZD; *n* = 42		*T*: 93%; *D*: 60%; *P*: 12%	Human study	Alleviate dysmenorrhoea Promote pregnancy	[27]
XZD; *n* = 42	Danazol; *n* = 28	*T*: 95.2% versus 75.0% *P* in XZD: 23.6%	Human study	Alleviate dysmenorrhoea Promote pregnancy	[28]
Xiaochaihu decoction (XCHD)	Danazol		SD rat model	COX-2↓ P450arom↓ Estradiol↓ IL-8↓ TNF-*α*↓ MVD↓ VEGF↓ Fas protein↑ apoptosis↑ in ectopic endometrial tissues	[29, 33–37]
Qu Yi Kang (QYK)	Danazol		SD rat model	TXB2↓ IL-2↓, IL-6↓ VEGF↓ ER↓	[39, 40, 42–44]
Yi Wei Ning (YWN); *n* = 46		*T*: 93.48%	Human study	Dysmenorrhoea alleviation Shrink endometriotic lesion	[45]
YWN			Wistar rat model	Fas/FasL↑ Bcl-2↓ Bax↑ apoptosis↑ COX-2↓ in ectopic endometrial tissues	[46, 48, 51]
Yi Wei San (YWS)	Danazol		Rabbit model	6-keto-PGF l*α*↓ TXB2↓ Blood rheology↑ Vasoactive substances↓	[51, 52]
Huoxue Xiaoyi decoction (HXD)			SD rat model	ICAM-1↓ MMP-9↓ VEGF↓ AAA pathway↓ Recurrence rate↓	[54, 55]

Note: *T* (Total effect rate) = all effective number of cases/total number of cases; effective case refers to the patients or animal models whose signs and symptoms have been improved after treatment; *D*: dysmenorrhoea alleviation rate; *P*: pregnancy rate.

**Table 4:** Chinese traditional patent medicine for EM treatment in literatures.

Chinese traditional patent medicine; sample/case number (*n*)	Control; sample/case number (*n*)	Total clinical effect rate: total effect rate (*T*)*; dysmenorrhoea alleviation rate (*D*); Pregnancy rate (*P*)	Model used	Therapeutic effects and actions	References
Guizhi Fuling Capsules; *n* = 40	Mifepristone Tablets; *n* = 40	*T*: 83.3% verus 87.5%	Human study	CA125↓ EMAb↓	[102]
Guizhi Fuling Capsules			SD rat model	CD+3↑ CD+4↑ CD+4/CD+8↑, CD+8↓	[103, 104]
Sanjie Zhentong Capsules; *n* = 112	Danazol; *n* = 46	*T*: 92.9 % verus 77.5%	Human study	Alleviate dysmenorrhoea Shrink endometrioic lesion	[105]
				Recurrence rate ↓	[106]
Yiweikang Granule; *n* = 50	Danazol; *n* = 50	*T*: 84.0% verus 86.0%	Human study	CA125↓ VEGF↓ E2↓ Alleviate dysmenorrhoea Shrink endometrioic lesion	[107, 108]
Dahuang Zhechong Wan; *n* = 26	Danazol; *n* = 15	*T*: 80.8% verus 86.7%	Human study	EMAb ↓ Alleviate dysmenorrhoea Shrink endometrioic lesion	[109]
Dahuang Zhechong Wan	Danazol		SD rat model	E2↓ PRL↓ P↑	[110]
Xiang Leng Wan; *n* = 54	Medroxyprogesterone Acetate Dispersible; *n* = 33		Human study	CA125↓ Alleviate dysmenorrhoea	[111]
Xiang Leng Wan	Danazol		SD rat model	IL-8↓ TNF-*α*↓	[112]

*Total effect rate (*T*) = all effective number of cases/total number of cases; effective case refers to the patients or animal models whose signs and symptoms have been improved after treatment.

**Table 5:** Main acupoints and minor acupoint for the treatment of endometriosis in reports.

The main acupoint	English name	*N*	Percentage	The minor acupoint	English name	*N*	Percentage
Guanyuan	RN4	46	30.67%	Zusanli	ST36	21	35%
Sanyinjiao	SP6	34	22.67%	Hegu	LI4	6	10%
Qihai	RN6	26	17.33%	Taichong	LR3	9	15%
Zhongji	RN3	17	11.33%	Shenshu	BL23	9	15%
Zigong	EX-CA1	11	7.33%	Wailing	ST26	4	6.67%
Xuehai	SP10	8	5.33%	Tianshu	ST25	5	8.33%
Diji	SP8	8	5.33%	Taixi	KI3	3	5%
				Guilai	ST29	3	5%

Note: *N*: total number of reports.
